# Light Intensity Modulates the Efficiency of Virus Seed Transmission through Modifications of Plant Tolerance

**DOI:** 10.3390/plants8090304

**Published:** 2019-08-27

**Authors:** Nuria Montes, Israel Pagán

**Affiliations:** 1 Fisiología Vegetal, Departamento Ciencias Farmacéuticas y de la Salud, Facultad de Farmacia, Universidad San Pablo-CEU Universities, 28668, Boadilla del Monte (Madrid), Spain and Servicio de Reumatología, Hospital Universitario de la Princesa, Instituto de Investigación Sanitaria (IIS-IP), 28006 Madrid, Spain; 2 Centro de Biotecnología y Genómica de Plantas UPM-INIA and E.T.S. Ingeniería Agronómica, Alimentaria y de Biosistemas, Departamento de Biotecnología-Biología Vegetal, Universidad Politécnica de Madrid, 28223 Madrid, Spain

**Keywords:** *Arabidopsis thaliana*, *Cucumber mosaic virus*, fecundity tolerance, light intensity, mortality tolerance, resistance, *Turnip mosaic virus*, virus seed transmission

## Abstract

Increased light intensity has been predicted as a major consequence of climate change. Light intensity is a critical resource involved in many plant processes, including the interaction with viruses. A central question to plant–virus interactions is understanding the determinants of virus dispersal among plants. However, very little is known on the effect of environmental factors on virus transmission, particularly through seeds. The fitness of seed-transmitted viruses is highly dependent on host reproductive potential, and requires higher virus multiplication in reproductive organs. Thus, environmental conditions that favor reduced virus virulence without controlling its level of within-plant multiplication (i.e., tolerance) may enhance seed transmission. We tested the hypothesis that light intensity conditions that enhance plant tolerance promote virus seed transmission. To do so, we challenged 18 *Arabidopsis thaliana* accessions with *Turnip mosaic virus* (TuMV) and *Cucumber mosaic virus* (CMV) under high and low light intensity. Results indicated that higher light intensity increased TuMV multiplication and/or plant tolerance, which was associated with more efficient seed transmission. Conversely, higher light intensity reduced plant tolerance and CMV multiplication, and had no effect on seed transmission. This work provides novel insights on how environmental factors modulate plant virus transmission and contributes to understand the underlying processes.

## 1. Introduction

Climate change is a multi-faceted phenomenon that entails increasing concentrations of greenhouse gases in the atmosphere (particularly CO_2_), rising temperatures, changes in precipitation patterns, and higher light intensity [1]. In the last decades, the rate at which climate change occurs has accelerated. Indeed, atmospheric CO_2_ is predicted to reach 730–1000 ppm by the year 2100, contributing to expected increases in global average surface temperature of 1.0–3.7 °C during this same time [1,2]. This global warming has been predicted to reduce cloud coverage, increasing light intensity [3], and promoting drought events [1]. Since these climatic factors influence most (if not all) biological processes, climate change is expected to have a huge impact in the reproductive success of living organisms and in the relationships that they establish e.g., [4,5]. Plant–virus interactions are not an exception [6,7], and therefore investigating the effect of climatic conditions in plant and viral fitness components is central to understand their outcome [7].

Upon virus infection, plant defenses are the main determinant of host fitness [8,9]. The two main host defenses against parasites are resistance, i.e., the host’s ability to limit parasite multiplication [10,11], and tolerance, i.e., the host’s ability to reduce the effect of infection on its fitness at a given parasite load [12,13]. Climate change conditions have been shown to affect plant resistance to viruses. For instance, elevated CO_2_ and temperature may enhance [14,15,16] or reduce [17,18] viral load. Similarly, light intensity and drought can also regulate the plant ability to control virus multiplication [19,20,21]. Comparatively much less is known on the effect of climatic factors on plant tolerance to viruses. Theoretical models on the evolution of tolerance predict that this defense strategy will be favored in environments with high resource availability, as there would be no limitation of the amount of nutrient/energy uptake needed to compensate fitness losses due to infection [22,23]. A critical resource for plants is light, which determines energy availability and controls central process such as germination, leaf proliferation, photosynthesis, bud and flower initiation, and cell division [24]. Many of these processes are linked to plant tolerance to viruses [9]. Thus, higher light intensity predicted by climate change models would promote plant tolerance to viruses. This hypothesis has been seldom experimentally tested [9,25], and how light intensity modulates plant tolerance to viruses remains largely unexplored.

On the other side of the interaction, virus reproductive success is determined by its ability to be transmitted to susceptible hosts. Recently developed mathematical models predict that on-going climate change will result in higher prevalence of infectious diseases [26,27]. These works proposed that variations in temperature and humidity will promote parasite plant-to-plant transmission (horizontal transmission) through effects on population dynamics of vectors that increase population sizes or biting rates [28,29,30,31]. This seems to be the case of plant viruses, many of which are transmitted by vectors. Indeed, lower humidity and higher temperature positively affect plant virus transmission by increasing vector reproductive success and flying activity, and by enhancing host competence [32,33,34]. Notably, mathematical models attribute to light intensity a minor role in virus horizontal transmission. However, transmission by vectors is far from being the only mode of plant virus dispersal. More than 25% of known plant viruses can be transmitted from-parent-to-offspring through seeds (vertical transmission) [35,36]. The fitness of vertically transmitted parasites is highly dependent on host reproductive potential, as hosts need to reproduce for the parasite to infect new individuals [37,38,39]. Hence, reduced virulence would favor plant virus seed transmission. Recently, the efficiency of seed transmission has been also associated with higher virus multiplication in reproductive organs [40]. Thus, environmental conditions that favor reduced virus virulence without controlling its level of within-plant multiplication (by definition, tolerance) may enhance vertical transmission. In this context, light may become a major determinant of virus transmission if increased light intensity results in higher tolerance as proposed by theoretical models [22,23]. However, to date this hypothesis has not been experimentally tested.

To test the hypothesis that higher light intensity enhances virus seed transmission through modifications of plant tolerance, we utilized *Turnip mosaic virus* (TuMV, *Potyviridae*) and *Cucumber mosaic virus* (CMV, *Bromoviridae*), and *Arabidopsis thaliana* (from here on “Arabidopsis”, Brassicaceae). Both viruses are commonly found in wild populations of Arabidopsis at up to 80% prevalence [41], indicating that the Arabidopsis–TuMV and Arabidopsis–CMV interactions are significant in nature. Tolerance to CMV and to TuMV varies across Arabidopsis accessions as a quantitative trait; and long-lived accessions with low seed production to total biomass ratio (Group 1 accessions) are generally more tolerant to CMV, but less tolerant to TuMV, than short-lived accessions that have high seed to biomass ratio (Group 2 accessions) [42,43,44]. Upon CMV infection, increasing light intensity has been shown to favor Arabidopsis tolerance, but this conclusion was based on analyses in a reduced number of plant accessions [25]. Interestingly, CMV [45,46] and TuMV [40] have been shown to be vertically-transmitted in Arabidopsis, the efficiency of seed transmission being associated with higher virus load in the plant inflorescence and with reduced virulence [40]. Thus, the selected experimental system provides an ideal opportunity to analyze the interaction between light intensity, plant tolerance to virus infection and the efficiency of seed transmission.

Herein, we quantify tolerance, considering both the effect of infection on plant progeny production (fecundity tolerance) and survival (mortality tolerance), and resistance of 18 Arabidopsis accessions to one CMV and two TuMV isolates at low and high light intensity. In these Arabidopsis accessions, and at both light conditions, we also measure the efficiency of seed transmission of the three virus isolates. Using this information, we address the following questions: (i) If light intensity modulates the efficiency of CMV and TuMV seed transmission, (ii) if light intensity affects Arabidopsis resistance and tolerance to CMV and TuMV, (iii) if plant resistance/tolerance is associated with the efficiency of virus seed transmission, and (iv) if these associations depend on light intensity.

## 2. Results

### 2.1. Effect of Light on CMV and TuMV Seed Transmission in Arabidopsis. 1

**Figure 1:**
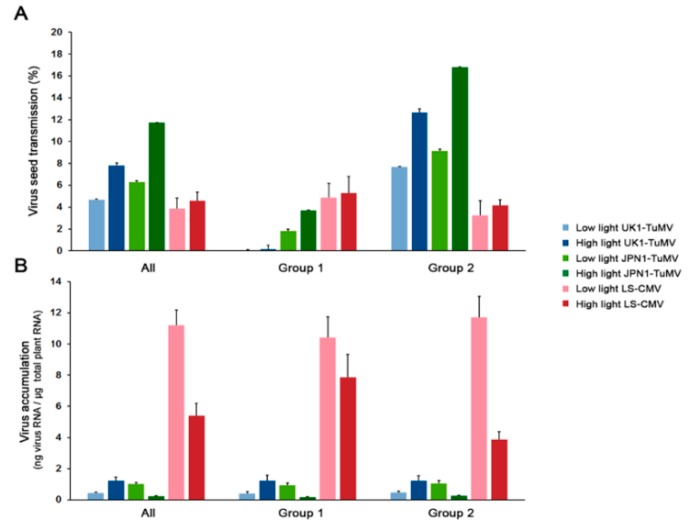
Effect of light intensity on: (**A**) The efficiency of virus seed transmission and (**B**) on virus accumulation. Values are mean ± standard error of 18 (All), 7 (Group 1) and 11 (Group 2) Arabidopsis accessions.

**Figure 2:**
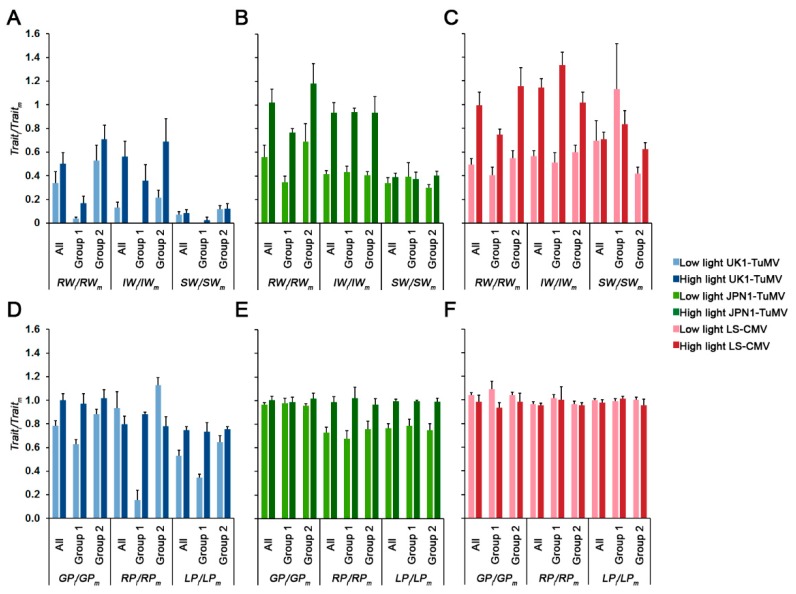
Effect of light intensity on Arabidopsis rosette weight (*RW*), inflorescence weight (*IW*) and seed weight (*SW*) when infected by UK1-TuMV (**A**), JPN1-TuMV (**B**) and LS-CMV (**C**); and effect of light intensity on Arabidopsis growth period (*GP*), reproductive period (*RP*) and life period (*LP*) when infected by UK1-TuMV (**D**), JPN1-TuMV (**E**) and LS-CMV (**F**). Values are mean ± standard error of 18 (All), 7 (Group 1) and 11 (Group 2) Arabidopsis accessions.

Per cent of seed transmission varied according to virus isolate, plant allometric group and light intensity (Wald *χ*^2^ ≥ 11.81, *p* ≤ 5 × 10^−4^). Overall, the efficiency of seed transmission was higher in JPN1-TuMV and lower in LS-CMV, with UK1-TuMV showing intermediate values (Wald *χ*^2^_1,859_ = 58.80, *p* < 1 × 10^−4^) (Figure 1A and Table S1). Hence, data for each virus isolate was also analyzed separately. UK1-TuMV seed transmission was higher at high light intensity (Wald *χ*^2^_1,287_ = 13.48, *p* = 2.4 × 10^−4^). Indeed, in both allometric groups, higher light intensity resulted in greater UK1-TuMV seed transmission (Wald *χ*^2^ ≥ 7.41, *p* ≤ 0.006) (Figure 1A). Also, UK1-TuMV seed transmission was higher in Group 2 than in Group 1 at both light intensities (Wald *χ*^2^ ≥ 7.901, *p* = 0.004). This difference was due to virus-induced plant castration of most Group 1 plants, as previously shown [43], which resulted in no seed production at low light intensity (and therefore no seed transmission) and very little at high light intensity (Figure 2A). JPN1-TuMV seed transmission was also generally higher at high light intensity (Wald *χ*^2^_1,290_ = 57.69, *p* < 1 × 10^−4^). Again, light intensity increased seed transmission in accessions of both allometric groups (Wald *χ*^2^ ≥ 6.71, *p* ≤ 0.010). JPN1-TuMV seed transmission was again higher in Group 2 than in Group 1 accessions at both light intensities (Wald *χ*^2^ ≥ 8.46, *p* ≤ 0.003). Finally, LS-CMV seed transmission was not affected by light intensity either when all accessions were analyzed together (Wald *χ*^2^_1,281_ = 2.52, *p* = 0.113), or when each allometric group was considered separately (Wald *χ*^2^ ≤ 0.42, *p* ≥ 0.515). Seed transmission was also similar in both allometric groups at both light conditions (Wald *χ*^2^_1,281_ = 1.47, *p* = 0.224) (Figure 1A). In summary, high light intensity increases TuMV, but not CMV, seed transmission.

### 2.2. Effect of Light on Arabidopsis Resistance to CMV and TuMV

The level of UK1-TuMV, JPN1-TuMV and LS-CMV RNA accumulation was used to evaluate Arabidopsis resistance to virus infection (Figure 1B). Generalized linear mixed models using virus isolate, Arabidopsis accession and light condition as factors indicated that virus accumulation varied according to the three factors (Wald *χ*^2^ ≥ 7.24, *p* ≤ 7 × 10^−3^), and to the interaction between virus and light intensity (Wald *χ*^2^_1,855_ = 11.94, *p* = 0.003). Thus, we analyzed the effect of light and allometric group on virus accumulation for each virus isolate separately. Light intensity affected the level of accumulation of the three viruses and in both allometric groups (Wald *χ*^2^ ≥ 21.47, *p* < 1 × 10^−4^): Higher light intensity increased UK1-TuMV accumulation, and reduced that of JPN1-TuMV and LS-CMV (Figure 1B). Exception were LS-CMV-infected Group 1 plants, for which light intensity did not affect virus multiplication (Wald *χ*^2^_1,106_ = 2.28, *p* = 0.131). On the other hand, no significant differences between allometric groups were observed in the accumulation of any of the three viruses at any light intensity (Wald *χ*^2^_1,281_ ≤ 1.29, *p* ≥ 0.256). Hence, light intensity affects one of the main infection traits associated with virus seed transmission [40].

### 2.3. Effect of Light on Arabidopsis Growth, Reproduction and Developmental Schedule upon CMV and TuMV Infection

Next, we analyzed if light intensity modulated how virus infection affected plant growth, reproduction and developmental schedule (Figure 2 and Table S1). For all three viruses, higher light intensity generally reduced the effect of virus infection on plant growth (effect of infection on rosette weight: *RW_i_*/*RW_m_*) and reproduction (effect of infection on inflorescence weight: *IW_i_*/*IW_m_*) (Wald *χ*^2^_1,281_ ≥ 14.33, *p* < 1 × 10^−4^). Similar results were obtained when each allometric group was analyzed separately (Wald *χ*^2^ ≥ 7.55, *p* < 6 × 10^−3^). At both light intensities, and for all viruses, the effect of infection on *RW* was higher in Group 1 than in Group 2 (Wald *χ*^2^ ≥ 4.17, *p* ≤ 0.041). Similar trends were observed for the effect of UK1-TuMV on *IW* (Wald *χ*^2^ ≥ 2.88, *p* ≤ 0.009), whereas for JPN1-TuMV no significant differences in *IW_i_*/*IW_m_* between allometric groups were observed at any light intensity (Wald *χ*^2^ ≤ 3.73, *p* ≥ 0.053). For LS-CMV, the effect on *IW* at low light intensity was higher in Group 1 plants, and the opposite was observed at high light intensity (Wald *χ*^2^_1,114_ = 34.38, *p* < 1 × 10^−4^) (Figure 2). For the three viruses, light had no effect on virus virulence (effect of infection on seed weight: *SW_i_*/*SW_m_*) when all Arabidopsis accessions were considered together (Wald *χ*^2^_1,281_ ≤ 3.78, *p* ≥ 0.066). However, when each allometric group was analyzed separately, higher light reduced UK1-TuMV virulence (higher *SW_i_*/*SW_m_*) in Group 1 plants (Wald *χ*^2^_1,106_ = 4.88, *p* = 0.027), and JPN1-TuMV and LS-CMV virulence in Group 2 plants (Wald *χ*^2^ ≥ 7.55, *p* < 6 × 10^−3^). At both light intensities, UK1-TuMV virulence was higher in Group 1 than in Group 2 (Wald *χ*^2^ ≥ 5.70, *p* ≤ 0.017), the opposite was observed for LS-CMV infected plants (Wald *χ*^2^ ≥ 8.36, *p* ≤ 0.004), and no differences between allometric groups were found in JPN1-TuMV-infected plants (Wald *χ*^2^ ≤ 0.25, *p* ≥ 0.619) (Figure 2A–C).

Light intensity also modified the effect of virus infection on plant developmental schedule (Figure 2D–F). For the two TuMV isolates, higher light intensity generally reduced the effect of virus infection on Arabidopsis growth period (*GP_i_*/*GP_m_*), reproductive period (*RP_i_*/*RP_m_*) and total life period (*LP_i_*/*LP_m_*), either when all accessions were considered together (Wald *χ*^2^_1,281_ ≥ 4.10, *p* ≤ 0.043), or when each allometric group was considered independently (Wald *χ*^2^ ≥ 4.60, *p* ≤ 0.032). Indeed, in many cases higher light intensity erased the effect of infection on plant development (trait ratios near 1). Exceptions were *RP_i_*/*RP_m_* in UK1-TuMV-infected plants, which showed the opposite trend (Wald *χ*^2^_1,281_ = 74.25, *p* < 1 × 10^−4^), and GP_i_/GP_m_ in Group 1 JPN1-TuMV-infected plants, for which no differences according to light intensity were observed (Wald *χ*^2^_1,106_ = 0.08, *p* = 0.773) (Figure 2D,E). On the other hand, light intensity increased the effect of LS-CMV infection on *GP* (Wald *χ*^2^_1,281_ = 13.47, *p* = 2 × 10^−4^), but not on *RP* and *LP* (Wald *χ*^2^_1,281_ ≤ 2.41 *p* ≥ 0.120). The observed effect on *GP* was due to an increase of the effect of infection at high light intensity in Group 1 plants (Wald *χ*^2^_1,106_ = 26.11, *p* < 1 × 10^−4^) (Figure 2F). No differences in the effect of virus infection on developmental traits was observed between allometric groups (Wald *χ*^2^ ≤ 1.06, *p* ≥ 0.302), except for UK1-TuMV-infected plants grown at low light intensity, for which *GP_i_*/*GP_m_*, *RP_i_*/*RP_m_* and *LP_i_*/*LP_m_* were larger in Group 2 than in Group 1 plants (Wald *χ*^2^ ≥ 66.72, *p* < 1 × 10^−4^) (Figure 2D–F). 

In summary, light intensity modulates the effect of infection on plant growth, reproduction and development. Previous work associated tolerance to TuMV with changes in the plant developmental schedule and that to CMV with modifications of resource reallocation from growth to reproduction [40,42]. Our results would be compatible with these previous works. Hence, we analyzed the effect of light intensity on plant tolerance to virus infection.

### 2.4. Effect of Light on Arabidopsis Tolerance to CMV and TuMV

**Figure 3:**
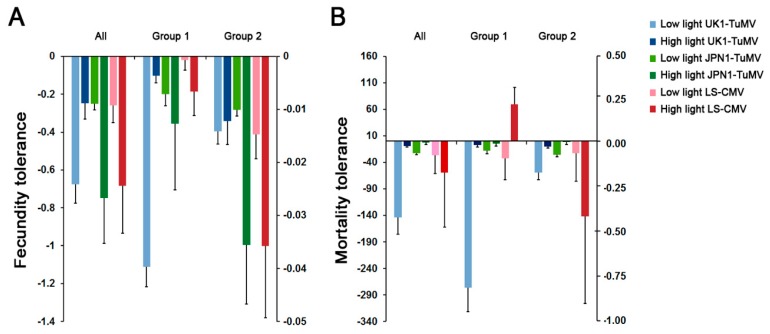
Arabidopsis fecundity and mortality tolerance to UK1-TuMV, JPN1-TuMV and LS-CMV. (**A**) Values of fecundity tolerance to virus infection measured as the slope of the *SW* to virus accumulation linear regression. (**B**) Values of mortality tolerance to virus infection measured as the slope of the *LP* to virus accumulation linear regression. Values are mean ± standard error of 18 (All), 7 (Group 1) and 11 (Group 2) Arabidopsis accessions.

Fecundity and mortality tolerances (slopes of the *SW* and *LP* to virus accumulation regression, respectively) differed depending on the virus isolate, the Arabidopsis accession and the light condition (Wald *χ*^2^ ≥ 4.51, *p* ≤ 0.032), the interaction between virus and the other two factors being significant (Wald *χ*^2^ ≥ 8.21, *p* ≤ 4 × 10^−3^) (Figure 3). Thus, we analyzed the effect of light on plant tolerance for each virus isolate separately. Light intensity increased (shallowed slope of the regression) Arabidopsis fecundity tolerance to UK1-TuMV (Wald *χ*^2^_1,34_ = 10.72, *p* = 0.001), which was due to the effect on Group 1 accessions (Wald *χ*^2^_1,12_ = 80.75, *p* < 1 × 10^−4^). At low light intensity, fecundity tolerance to UK1-TuMV infection was higher in Group 2 than in Group 1 accessions (Wald *χ*^2^_1,18_ = 36.303, *p* < 1 × 10^−4^). Light intensity decreased (steeper slope of the regression) Arabidopsis fecundity tolerance to JPN1-TuMV (Wald *χ*^2^_1,34_ = 4.22, *p* = 0.040). Here, this was due to the effect on Group 2 accessions (Wald *χ*^2^_1,21_ = 5.19, *p* = 0.022). Similar results were obtained for fecundity tolerance to LS-CMV (Wald *χ*^2^ ≤ 5.36, *p* ≥ 0.027). For both JPN1-TuMV and LS-CMV, fecundity tolerance was higher in Group 1 than in Group 2 accessions at both light intensities (Wald *χ*^2^ ≥ 5.41, *p* ≤ 0.020). Mortality tolerance to both TuMV isolates was higher at higher light intensity either when all accessions were considered together (Wald *χ*^2^ ≥ 17.58, *p* < 1 × 10^−4^), and when each allometric group was analyzed separately (Wald *χ*^2^ ≥ 11.67, *p* < 1 × 10^−4^). For LS-CMV, the effect of viral load on plant mortality was minimal, as none of the LP to virus accumulation slopes significantly differed from zero (Wald *χ*^2^ ≤ 0.48, *p* ≥ 0.489). These near-zero slopes were observed at both light intensities, indicating that this factor did not affect mortality tolerance to LS-CMV (Figure 3). Hence, light intensity changed fecundity tolerance to the three viruses but only affected mortality tolerance to TuMV. 

### 2.5. Relationship between Light Intensity, Virus Seed Transmission and Plant Tolerance to CMV and TuMV

**Figure 4:**
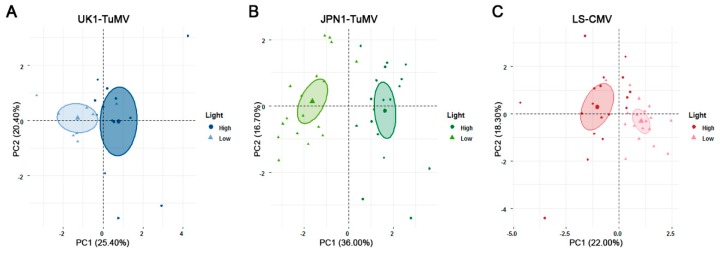
Principal component analysis of data for the effect of UK1-TuMV (**A**), JPN1-TuMV (**B**) and LS-CMV (**C**) infection on the plant developmental schedule, growth and reproduction traits, virus accumulation, fecundity and mortality tolerance and virus seed transmission from 18 accessions of Arabidopsis grown under high (circle) and low (triangle) light conditions. Colored ellipses represent 95% confidence intervals around the mean (bold point) for each light intensity.

The analyses above indicated that light intensity modulates the efficiency of seed transmission, as well as virus multiplication, virulence and plant tolerance to infection. Thus, we analyzed the interplay between these virus, host and environmental traits. To do so, we performed Principal Component Analyses (PCA) using all the measured traits in order to explain the variance in the outcome of virus infection observed at different light intensities. These PCA analyses were done for each virus isolate separately, and considered mean values of all accessions together as in general light similarly affect the performance of both allometric groups (Figure 4).

For UK1-TuMV, the PCA yielded two major Principal Components (PCs) that together explained about half of the total variance (Table S2). Virus accumulation and efficiency of seed transmission, plant mortality tolerance, and the effect of infection on *RP* and *LP* loaded into PC1 (*r* = −0.66 to 0.73; *p* ≤ 0.008), whereas the effect of infection on *GP*, *RW* and *IW*, and fecundity tolerance loaded into PC2 (*r* = −0.77 to 0.73; *p* ≤ 0.029). Interestingly, PC1 separated the performance of accessions at low and at high light intensity upon virus infection (Figure 4A). Thus, the difference in the outcome of UK1-TuMV infection can be explained by the joint effect of seed transmission efficiency, virus accumulation and mortality tolerance. Note that the loading of these traits into the same PC indicates that they are associated. Moreover, the three variables loaded with the same sign into the PC, meaning that higher virus accumulation and mortality tolerance is associated with increasing efficiency of seed transmission. The PCA for JPN1-TuMV-infected plants yielded again two major PCs jointly explaining 53% of the total variance (Table S2). The effect of infection on *IW*, *RP* and *LP*, plus virus accumulation and efficiency of seed transmission, and plant mortality tolerance loaded into PC1 (*r* = −0.71 to 0.82; *p* ≤ 0.021). Virus virulence, effect of infection on *GP* and plant fecundity tolerance loaded into PC2 (*r* = −0.63 to 0.76; *p* < 1 × 10^−4^). Again, PC1 allowed distinguishing between plant performance at low and high light intensity, indicating that this difference can be explained by the joint effect of seed transmission efficiency, virus accumulation and mortality tolerance (Figure 4B). Finally, the PCA using the data of LS-CMV-infected plants yielded two main PCs that explained 40% of the total variance (Table S2). Virus accumulation and virulence, plant fecundity tolerance, and the effect of virus infection on *RW* and *IW* loaded into PC1 (*r* = −0.76 to 0.76; *p* < 1 × 10^−4^). Plant mortality tolerance and the effect of infection on *GP* loaded into PC2 (*r* = −0.71 to 0.84; *p* ≤ 0.029). In this case, the efficiency of seed transmission loaded into PC3 (*r* = 0.57; *p* = 3 × 10^−4^), which explained 15% of the total variance. The highest discriminative power of the plant performance according to light intensity was achieved combining PCs 1 and 2 (Figure 4C), with PC3 having little discriminative power (Table S2). Therefore, for LS-CMV-infected plants tolerance and virus multiplication are not associated with seed transmission, the latest trait having no role in explaining plant performance at different light intensities.

Together, these results indicate that the effects of light intensity on the TuMV-Arabidopsis interaction are explained by the combined modifications of TuMV seed transmission, virus multiplication and mortality tolerance, which are positively associated between them.

## 3. Discussion

Accelerating rates of climate change are predicted to have an enormous impact on the relationships between organisms [5], including those established by causal agents of plant infectious diseases [7]. In this context, understanding how environmental conditions affect parasite transmission is key to understand the emergence of plant disease epidemics, as the ability to spread to new susceptible hosts is a major determinant of parasite fitness [37,47]. Notably, very little is known about how environmental cues affect plant parasite transmission, particularly for plant viruses. Moreover, most of the work on this subject focused on horizontal transmission through vectors [48], largely neglecting other major modes of virus dispersal such as vertical transmission through seeds [35]. Here, we provide evidence that light intensity (one of the environmental factors predicted to change due to climate change) affects the efficiency of virus seed transmission in a species-dependent manner, and that this change is associated with modifications of plant defenses.

Our results indicate that higher light intensity increases the efficiency of TuMV seed transmission in Arabidopsis. Virus seed transmission has a high impact in plant virus epidemiology [35,46]. Seed infection provides the virus with a mean to persist for long periods of time when hosts or vectors are not available, as many seed transmitted viruses can survive within the seed as long as it remains viable [35,49]. Seed transmission allows also for long distance dissemination of the virus via infected seeds, as seeds can travel further than most virus vectors [50]. However, perhaps the most important epidemiological effect of seed transmission is that it represents an important source of primary inoculum: Many viruses with this mode of transmission can be also horizontally transmitted, such that viruses that infect plants through seeds can be disseminated afterwards via plant-to-plant contact or by insect vectors [35,36,51]. Therefore, our results would be compatible with higher light intensity acting as a factor that favor virus epidemics. This is in line with analyses of the effect of changes in other environmental factors that entails climate change. For instance, it has been shown that higher temperature often promotes virus seed transmission [reviewed by 35]. At odds, CMV seed transmission was not affected by light intensity. Interestingly, to our knowledge the only other analysis of how climatic conditions affect CMV vertical transmission showed that drought (another predicted consequence of global warming) decreased seed transmission in lupin [52]. Taken together, these observations suggest that climate change may favor seed transmission for certain viruses, but does not seem to be a general trend.

Light intensity also affected plant defenses. First, higher light intensity reduced resistance to UK1-TuMV and increased that to JPN1-TuMV. Interestingly, it has been nicely shown that both isolates induce different responses in Arabidopsis, including differential expression of genes involved in the immune and defense responses [53]. In addition, sequence comparison of the UK1- and JPN1-TuMV genomes revealed that the P3 protein, which is involved in host resistance, is the most divergent region at the amino acid level [53]. These differences between UK1- and JPN1-TuMV infection in Arabidopsis may explain the differential effect of light intensity on plant resistance. Higher light intensity also increased plant resistance to LS-CMV, which is in agreement with previous observations [25]. Second, light intensity also modulated Arabidopsis fecundity and mortality tolerance to virus infection. Higher light intensity increased fecundity tolerance to UK1-TuMV, and reduced that to JPN1-TuMV and LS-CMV. Arabidopsis fecundity tolerance to UK1-TuMV has been associated with modifications of the plant developmental schedule: The larger the life period, the higher the fecundity tolerance [54]. In agreement, our results indicate that higher light intensity increases the life period of plants infected by UK1-TuMV of both allometric groups. Moreover, this increase is smaller in Group 2 than in Group 1 plants, which is accompanied by a lesser effect of light on plant fecundity tolerance in the latter group of accessions. On the other hand, fecundity tolerance to JPN1-TuMV and LS-CMV has been associated with resource reallocation from growth to reproduction. This resource reallocation was denoted by a higher effect of infection on *RW* than on *SW* [42,54]. Our results indicate that higher light intensity prevents such resource reallocation (i.e., the effect of infection on *SW* becomes greater than on *RW*). This could explain the reduction of fecundity tolerance to JPN1-TuMV and LS-CMV at higher light intensity. These observations are at odds with previous analyses in the same plant-virus interaction showing that higher light intensity promotes fecundity tolerance [25]. However, these authors used only four Arabidopsis genotypes (two per allometric group). More importantly, they estimated point fecundity tolerance (the effect of infection at a given pathogen load), rather than range fecundity tolerance (the slope of a regression of host fitness against pathogen load) as measured here; and it has been shown that these two measures may lead to different conclusions [9,12]. In contrast with fecundity tolerance, both TuMV isolates showed increased mortality tolerance under high light conditions. Note that plants infected by TuMV, but not by CMV, generally increased *GP* and *RP* in high light conditions, which would explain the increase in mortality tolerance to TuMV. 

Our analyses on how light intensity modifies plant defenses may offer a potential mechanism for the differential effect of light intensity on TuMV and CMV seed transmission. PCA analyses showed that the main PC differentiating the performance of TuMV-infected plants under high and low light conditions (PC1) contained seed transmission efficiency, virus accumulation and mortality tolerance as the main contributors. These three traits loaded positively onto the PC, meaning that high light conditions increased their values. In accordance, high and low light conditions were clearly differentiated into the positive and negative regions of PC1, respectively. The loading of seed transmission efficiency, virus accumulation and mortality tolerance into the same PC indicated that these variables were correlated. Therefore, higher efficiency of seed transmission at high light intensity was associated with higher virus multiplication and plant mortality tolerance. It has been proposed that higher virus multiplication in the plant reproductive structures favors embryo/gametophytes invasion by promoting the virus crossing of the boundary between the maternal and progeny tissues [55]. The association between increasing virus multiplication at high light intensity and higher per cent of infected seeds is in line with this prediction, and is also in agreement with previous analyses that identified within-host virus multiplication as a key determinant of seed transmission [40]. In addition, our analyses provide support for the hypothesis that higher (mortality) tolerance to TuMV infection promotes seed transmission. Interestingly, the effect of infection on *RP* and *LP*, both associated with mortality tolerance, also loaded positively onto PC1, indicating that these traits are also positively associated with seed transmission efficiency. We have recently identified the speed of within-host movement as a major determinant of the efficiency of seed transmission [40]: Faster within-host movement increases the virus chances for reaching the plant reproductive structures, which in turn favors seed transmission [56]. Similarly, larger *RP* associated with higher mortality tolerance may expand the time span for the virus to reach reproductive organs. Note that high light intensity did not increase JPN1-TuMV multiplication, which suggest that mortality tolerance is the predominant factor explaining the interplay between light and seed transmission. Accordingly, for plants infected with both TuMV isolates mortality tolerance had the highest contribution to PC1. In contrast to TuMV, PCA of CMV-infected plants showed that virus accumulation, efficiency of seed transmission and plant tolerance loaded each on a different PC, indicating the lack of correlation between these three traits. In this case, seed transmission loaded into PC3, which could not discriminate between plant performance at high and low light intensity. This is in agreement with the lack of effect on this environmental condition on CMV seed transmission, and with the negative effect of high light on virus multiplication and plant tolerance.

In summary, our results show that light intensity affects the efficiency of seed transmission, a mode of dispersal that is common to more than a quarter of all known plant viruses. We also present evidence that increased efficiency of virus seed transmission at high light intensity is associated with environment-related modifications of plant resistance and tolerance. Hence, this work provides novel insights on the potential of climate change conditions to promote the dispersal of plant viruses by modifying the outcome of plant-virus interactions, and contributes to understand the underlying processes.

## 4. Materials and Methods

### 4.1. Arabidopsis Accessions and Virus Isolates

Virus isolates UK1-TuMV (Acc.N. AB194802), JPN1-TuMV (Acc.N. KM094174), Fny-CMV (Acc.N. NC_002034, NC_002035 and NC_001440, LS-CMV (Acc.N. AF416899, AF416900 and AF127976) and De72-CMV (not sequenced) were used. JPN1-TuMV was obtained from a field-infected plant of *Raphanus sativus* (Brassicaceae) [57] and De72-CMV from a field-infected plant of *Diplotaxis erucoides* (Brassicaceae) [58], and both were propagated in *Nicotiana benthamiana* plants. UK1-TuMV, Fny-CMV and LS-CMV were derived from biologically active clones [59,60,61] by in vitro transcription with T7 RNA polymerase (New England Biolabs, Ipswich, USA), and transcripts were used to infect *N. benthamiana* plants for virus multiplication.

Eighteen Arabidopsis accessions were used (Table 1). Ten accessions represented the Eurasian geographic distribution of the species and the remaining eight represented its distribution in the Iberian Peninsula, a Pleistocene glacial refuge for Arabidopsis [62]. Plant seeds were surface-sterilized (see below) and stratified for seven days at 4 °C in pots of 15 cm of diameter, 0.43 L volume containing 3:1, peat:vermiculite mix. Afterwards, pots were moved for seed germination and plant growth to a greenhouse at 22 °C, under 16 h light. Plants were mechanically inoculated, either with *N. benthamiana* TuMV- and CMV-infected tissue ground in 0.1 M Na2HPO4 + 0.5 M NaH2PO4 + 0.02% DIECA, or with inoculation buffer for mock-inoculated plants. Inoculations were done when plants were at developmental stages 1.05–1.06 [63]. After inoculation, plants were placed in two greenhouse modules: One with light intensity of 120–150 mol s/m^2^ (low light), and the other with light intensity of 250–300 mol s/m^2^ (high light). These conditions were chosen such that simulated light as a limiting factor [low light, 54] and no light limitation [high light, 25]. In both light conditions, plant accessions conformed two allometric groups as previously described [25]. Since plant allometry has been repeatedly reported as a relevant factor to understand Arabidopsis tolerance to virus infection [9], allometric group was considered as a factor in all analyses. For each Arabidopsis accession and light condition, seven to ten plants per virus were inoculated, and other seven were mock inoculated. All individuals were randomized in the greenhouse.

### 4.2. Virus Multiplication

TuMV and CMV multiplication were quantified as viral RNA accumulation via qRT-PCR in each individual plant. Virus accumulation was quantified from three disks of 4 mm in diameter collected from different systemically infected leaves. Form these plant samples, total RNA extracts were obtained using TRIzol^®^ reagent (Life Technologies, Carlsbad, CA, USA), and 10 ng of total RNA were added to the Brilliant III Ultra-Fast SYBR Green qRT-PCR Master Mix (Agilent Technologies, Santa Clara, CA, USA) according to manufacturer’s recommendations. Specific primers were used to amplify a 70 nt fragment of the TuMV, and a 106 nt fragment of the CMV, coat protein (CP) gene, respectively [64,65]. Each plant sample was assayed by duplicate on a Light Cycler 480 II real-time PCR system (Roche, Indianapolis, IN, USA). Absolute viral RNA accumulation was quantified as ng of viral RNA per μg of total RNA utilizing internal standards. For TuMV, internal standards consisted in ten-fold dilution series of plasmid-derived RNA transcripts of the same 70nt CP fragment from UK1-TuMV. For CMV, ten-fold dilution series were prepared using purified viral RNA. All internal standards ranged from 2 × 10^−3^ ng to 2 × 10^−7^ ng.

### 4.3. Effect of Infection on Plant Growth, Reproduction and Development

Aboveground plant structures were harvested at complete senescence and dry weight was determined after maintaining plants at 65 °C until constant weight. The weights of the rosette (*RW*), inflorescence (*IW*), and seeds (*SW*), were obtained. *RW* was used as an estimate of plant resources dedicated to growth, and *IW* was taken as an estimate of plant resources dedicated to reproduction [66]. The effect of virus infection on these traits was quantified by calculating infected to mock-inoculated plants ratios for each of them, dividing the value of each infected plant by the mean value of the mock-inoculated plants of the same genotype (*Trait_i_*/*Trait_m_, i* and *m* denoting infected and mock-inoculated plants, respectively). Virulence was estimated as ratio between infected and mock-inoculated plants of seed weight (*SW_i_*/*SW_m_*).

Three plant life-history traits were recorded: Growth period (*GP*), as days from inoculation to the opening of the first flower; and reproductive period (*RP*), as days from the opening of the first flower to the shattering of the first silique. In Arabidopsis, the opening of the first flower co-occurs with the end of the rosette growth, and the shattering of the first silique co-occurs with the end of flower production [63]. The total life period (*LP*), as time to plant senescence, was also quantified. 

### 4.4. Arabidopsis Tolerance

Fecundity and mortality tolerances of each Arabidopsis genotype were calculated as the slope of the linear regression of *SW* and *LP*, respectively, to virus accumulation considering both infected and mock-inoculated plants [12,13]. Importantly, seed viability, estimated as per cent germination, did not significantly differ between mock-inoculated and infected plants (*χ*^2^ ≤ 3.12; *p* ≥ 0.102). Also, virus infection did not affect the weight of a single seed (Wald *χ*^2^ ≤ 1.21; *p* ≥ 0.137). Thus, *SW* similarly reflects the number of viable seeds in both mock-inoculated and infected plants.

### 4.5. Efficiency of Virus Seed Transmission

The efficiency of virus seed transmission was estimated as per cent of infected seeds that gave rise to infected progeny per plant. Accordingly, we measured virus seed transmission to seedlings. For each replicate, 100 seeds were washed in a 10% household bleach solution (4% active chlorine) to ensure that any viral infection that occurred was not simply the result of virus presence on the seed coat, but rather the result of embryonic infection. Seeds were kept in this solution for 5 min and washed three time in sterile distilled water. Then, seeds were placed in Petri dishes containing Murashige and Skoog medium, stratified for three days at 4 °C, and germinated in a growth chamber at 22 °C, under 16 h light (intensity: 120–150 mol s/m^2^). Following [67], fifteen days post-stratification seedlings were pooled in groups of three for a total of 33 groups per replicate. These groups were tested for TuMV or CMV via qRT-PCR as above. Since we knew the proportion of samples that tested negative, we used a Poisson distribution to estimate the probability that more than one seedling would test positive in the same sample. Per cent of virus-infected seeds (*ST*) was then estimated using the expression reported by [68], $p=1-{\left(1-\frac{y}{n}\right)}^{\frac{1}{k}}$, where *p* is the probability of virus transmission by a single seed, *y* is the number of positive samples, *n* is the total number of samples assayed (*n* = 33), and *k* is the number of seedlings per sample (*k* = 3).

### 4.6. Statistical Analyses

With the exception of *RP_i_*/*RP_m_*, all analyzed traits were not normally distributed, and variances were heterogeneous according to Kolmogorov-Smirnoff and Levene’s tests, respectively. *SW_i_*/*SW_m_*, *IW_i_*/*IW_m_* and *ST* were n + 1 log-transformed. Therefore, differences in each trait between light intensities, viruses and allometric groups were analyzed by Generalized Linear Mixed Models (GzLMMs). The transformed and the rest of untransformed traits were fitted to a Log-normal distribution according to Akaike’s Information Criteria (AIC), with the exception of *RP_i_*/*RP_m_* that was fitted to a Gaussian distribution (R package: rriskDistributions, [69]). Light intensity was considered as a fixed factor, and Arabidopsis allometric group and virus were considered as random factor. GzLMMs were performed using R-libraries lme4, nlme and lmerTest [70,71,72]. 

To describe the relation between the effect of infection on the plant developmental schedule, growth and reproduction traits, virus accumulation, fecundity and mortality tolerance and virus seed transmission a Principal Component Analysis (PCA) was done for each virus. PCAs were performed using mean values per accession, which were scaled to zero mean and unit variance, inserted in a regression matrix and rotated using Varimax to obtain the Principal Components (PCs) using R-libraries FactorMiner and factoextra [73,74]. Statistical analyses were conducted using R version 3.6.1 [75].

## Figures and Tables

**Table 1:** Arabidopsis accessions used in this work, their geographical origin and allometric group.

Accession	Origin	Allometric Group
An-1	Amberes (Belgium)	Group 2
Bay-0	Bayreuth (Germany)	Group 2
Cad-0	Candelario (Spain)	Group 1
Cdm-0	Caldas de Miravete (Spain)	Group 1
Cen-1	Centenera (Spain)	Group 2
Col-0	Columbia (Unknown)	Group 2
Cum-0	Cumbres Mayores (Spain)	Group 1
Cvi	Cape Verde Islands	Group 2
Fei-0	Santa María da Feira (Portugal)	Group 2
Kas-0	Kashmir (India)	Group 1
Kas-2	Kashmir (India)	Group 1
Kyo-1	Kyoto (Japan)	Group 1
L*er*	Landsberg (Poland)	Group 2
Ll-0	Llagostera (Spain)	Group 1
Mer-0	Mérida (Spain)	Group 2
Pro-0	Proaza (Spain)	Group 2
Shak	Shakdara (Tadjikistan)	Group 2
Ver-5	Verin (Spain)	Group 2

## Supplementary Materials

The following are available online at https://www.mdpi.com/2223-7747/8/9/304/s1, Table S1: TuMV and CMV infection parameters measured in the 18 Arabidopsis accessions, Table S2: Results of Principal Component Analyses for the performance of Arabidopsis upon virus infection at different light intensities.
